# Comparative analysis of two methods in circumcision: a new disposable device versus classic sleeve technique

**DOI:** 10.1186/s12894-024-01513-9

**Published:** 2024-06-14

**Authors:** Sinan Kılıç

**Affiliations:** Department of Pediatric Surgery, Private Gebze Yuzyil Hospital, Clinic of Pediatric Surgery, Gebze, Kocaeli Turkey

**Keywords:** Circumcision, Clamp, Ring devices, Children, Suturless

## Abstract

**Purpose:**

Circumcision is the most common surgical procedures performed in males. Medical circumcision is recommended for diseases such as phimosis, paraphimosis, balanoposthitis and common urinary tract infections, although there is no exact indication. Conversely, Jewish and Muslim individuals commonly undergo circumcision regardless of medical necessity. Circumcision devices are designed to shorten surgery time, achieve an aesthetic appearance and ensure safe surgery. The aim of this study is to evaluate the effectiveness of the NeoAlis clamp, a disposable circumcision device, by comparing it with the sleeve technique in children.

**Materials and methods:**

Between 2017 and 2023, retrospective evaluation of 2626 patients who underwent circumcision using either the NeoAlis clamp (group 1) or the sleeve technique (group 2) was conducted. Operation time, results, cost, complications were compared between the two groups.

**Results:**

The study encompassed 2626 patients who fulfilled the inclusion criteria. Group 1 comprised 2403 patients, whereas Group 2 consisted of 223 patients. The overall complication rate, as denoted by *n* = 47, was 1.7%. Group 1 operation time was shorter than group 2. Bleeding, the most feared complication in the early period, was higher in the second group. No statistically significant difference was observed between the two groups regarding cost comparison.

**Conclusion:**

The primary concern during circumcision is to avoid complications related to general anesthesia in newborns and infants. The use of disposable ring devices has been facilitated by the shorter operation time and the absence of the need for sutures when performing circumcision under local anesthesia. However, knowledge of advanced surgical circumcision techniques is necessary in cases of bleeding and inappropriate ring placement.

**Supplementary Information:**

The online version contains supplementary material available at 10.1186/s12894-024-01513-9.

## Introduction

Circumcision is common surgical procedure in males often practiced in various countries due to religious and cultural traditions, especially as routine newborn and infant circumcision [1]. It is estimated that approximately one-third of men worldwide underwent this procedure. Dating back approximately 15,000 years, circumcision initially had cultural, ritualistic, and religious motives, with medicalization beginning in the 19th century. The earliest written evidence of circumcision dates back to 2300 B.C. in Ancient Egypt’s Ankh-Mahor temple wall reliefs [2].

Routine circumcision is discussed all over the world. Proponents cite its benefits, including improved hygiene and reduced risk of sexually transmitted infections (STIs). Additionally, they argue for its potential to decrease the risk of penile and cervical cancers, although evidence for this association is weak [3]. Conversely, opponents dispute or downplay these advantages, highlighting significant complication rates and potential reduction in penile sensation [4, 5]. Moreover, studies have shown that getting circumcised can lower the chances of getting HIV by up to 60%. The World Health Organization (WHO) and the Joint United Nations Program on HIV/AIDS (UNAIDS) support adult circumcision as well as circumcision for newborns as long-term ways to prevent HIV [6].

Various surgical techniques have been described for circumcision throughout history. Today, these methodologies are generally divided into two main approaches: circumcision with devices and classical surgical circumcision. Example of the most commonly used methods: Gomco clamp, Mogen clamp, PrePex device, Plastibell, NeoAlis clamp, Bone cutter method, Dorsal slit (open cut) method and Sleeve technic [7].

Circumcision has complications like all surgical procedures. These typically include early complications such as leakage hemorrhage, wound infection, pain, and swelling. However, more serious problems such as prolonged bleeding and amputation of the glans penis may also be encountered [8]. Long-term complications include persistent pain, wound infections, mucosal and skin adhesions, meatal stenosis, fistulas, decreased penile sensitivity, and changes in sexual function [9].

While sleeve circumcision represents a well-established and effective traditional approach with various adaptations evolving over time, the NeoAlis clamp, characterized by its disposable plastic design, presents a rapid and reliable method for performing circumcision procedures [10–14].

The hypothesis of this study is that circumcision performed with disposable clamps under local anesthesia in newborns and infants will result in shorter surgery time and fewer complications than the classical surgical technique. This study aims to compare the operative time, costs and complications associated with circumcision procedures using the NeoAlis clamp and sleeve technique.

## Materials and methods

### Study design

This study, following approval from the Ethics Committee of Istanbul Medipol University and the Institutional Review Board (Approval No. 2024/02/09), involved a retrospective review of data from 2626 children who underwent circumcision at our hospital between January 2017 and December 2023. Consent for publication was obtained from the family of the patient. The review conducted by a pediatric surgeon excluded patients with specific conditions such as hypospadias, epispadias, micropenis, disorders of sex development (DSD) and prematurity, resulting in a total of 2626 patients. These patients were categorized into two groups based on the circumcision method used: group 1 underwent circumcision using the NeoAlis Clamp (Patent No. 2011/06553, Turkish Patent and Trademark Office, Aba Group Healthcare Company, Ankara, Turkey), while group 2 underwent the sleeve technique. The anesthesia and circumcision method were determined based on patient age.

### Study population

In Group 1, circumcision for newborns and infants who under 12 months was performed using local anesthesia with the “NeoAlis clamp” method. This choice aimed to minimize the risk of neurodegenerative damage associated with general anesthesia and was also preferred by families who opted against general anesthesia during the procedure. For children over 12 months old in Group 2, circumcision was conducted under general anesthesia to mitigate potential psychological trauma due to their developed consciousness. Additionally, the reduced risk of neurodegenerative damage associated with general anesthesia after one year of age influenced this decision [15, 16]. Circumcisions for this group were performed using the sleeve technique under general anesthesia.

Circumcision performed using devices such as clamps simplifies and makes the procedure faster by reducing steps such as suturing and bleeding control. This faster approach is ideal for newborn and infant patients who have difficulty cooperating under local anesthesia. Additionally, for infants under one year of age, cuffing helps prevent excessive scarring from stitches due to the thinner foreskin. However, the clamp may cause discomfort in older children, especially those over five years of age who are more conscious and may find it uncomfortable to wear the device for close to a week.

### Surgical technique

#### NeoAlis Clamp

**Fig. 1:**
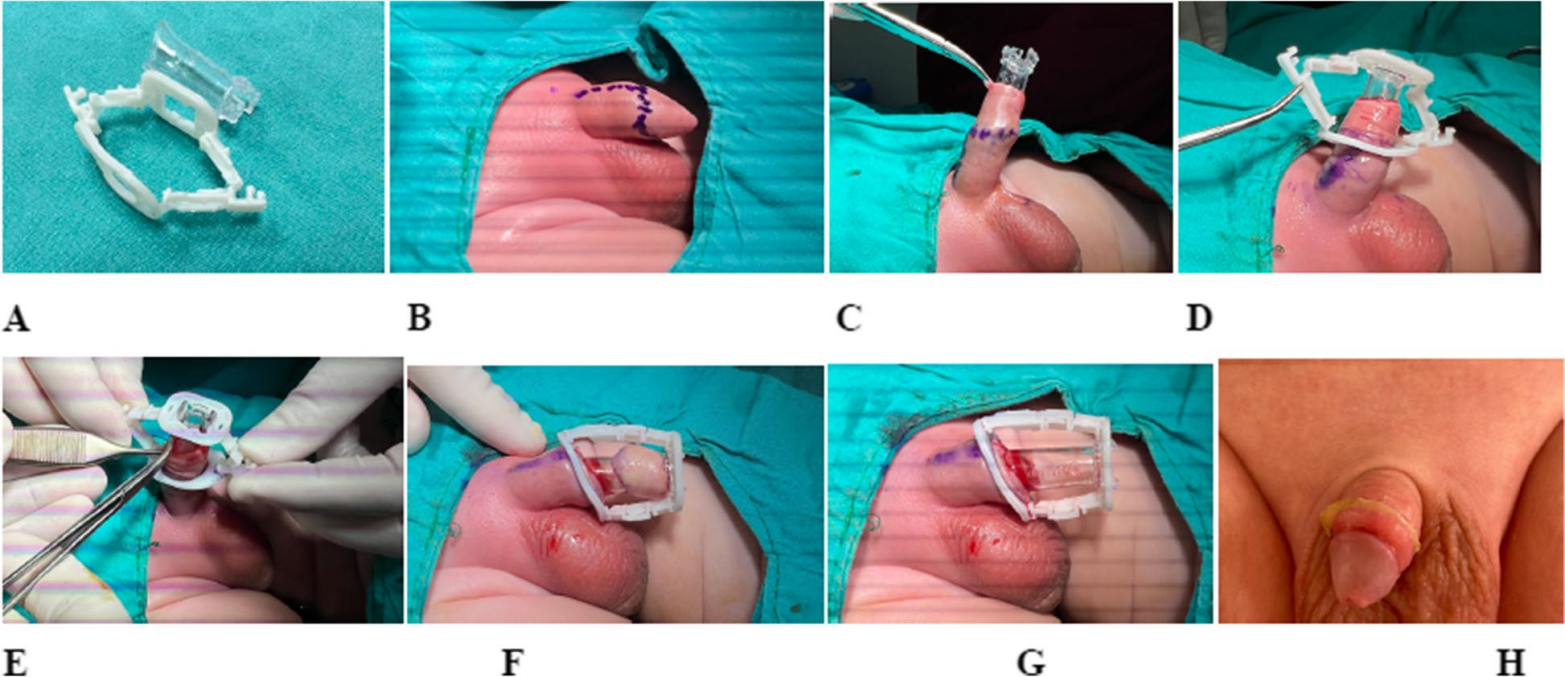
Steps of circumcision with NeoAlis clamp. **A** The appearance of the Neo Alis clamp consists of two parts; the transparent glans protector and the white ring, which serves as the compression device. **B** Marking the prepuce to be removed is crucial to avoid iatrogenic tortipenis formation. **C** By opening the prepuce edges with a clamp, the transparent glans protector device is placed. **D** Following the transparent device, the compression apparatus is attached, leaving the prepuce between the two devices. **E** This is the most crucial step, where the inner mucosa is shortened by pulling it upwards with the help of a clamp and forceps to avoid excessive length. **F** After the compression process, the prepuce portion remaining distal to the ring is removed. **G** Once the prepuce is removed, the procedure is completed. **H** Appearance one week after the removal of the Neo Alis clamp

The study excluded patients who requested general anesthesia. Prior to the penile ring block, lidocaine cream (EMLA, Akorn Pharmaceuticals, IL, USA), which contains lidocaine and prilocaine, was applied 45 min in advance, covering the penile base, skin, and prepuce. Local anesthesia was initiated with the penile ring block technique as needed at the beginning of all procedures. Lidocaine (Jetokain Simplex, Adeka Therapeutics, Istanbul, Turkey) was used at concentrations up to 20 mg per milliliter, with dosage adjustments based on patient weight. After dissection of preputial adhesions, the coronal sulcus behind the glans penis was fully exposed. Markings were made at the 12 and 6 o’clock positions with the prepuce pulled to a neutral position. Projections of the coronal sulcus were marked by retracting the penile skin, which is crucial for estimating erect penile skin length. An inner tube was inserted into the prepuce, with a dorsal slit made if necessary. The outer chamber was then unlocked, and the inner mucosa was tailored with trans-illumination guidance. Locked arms were positioned at the 6 and 12 o’clock positions, and an incision was made while leaving a 2- to 3-mm safety margin over the locked chamber. The prepuce was divided longitudinally and excised using a NeoAlis clamp, as shown in Fig. 1. (Video 1. Circumcision with Neo Alis Clamp.)

**Fig. 2:**
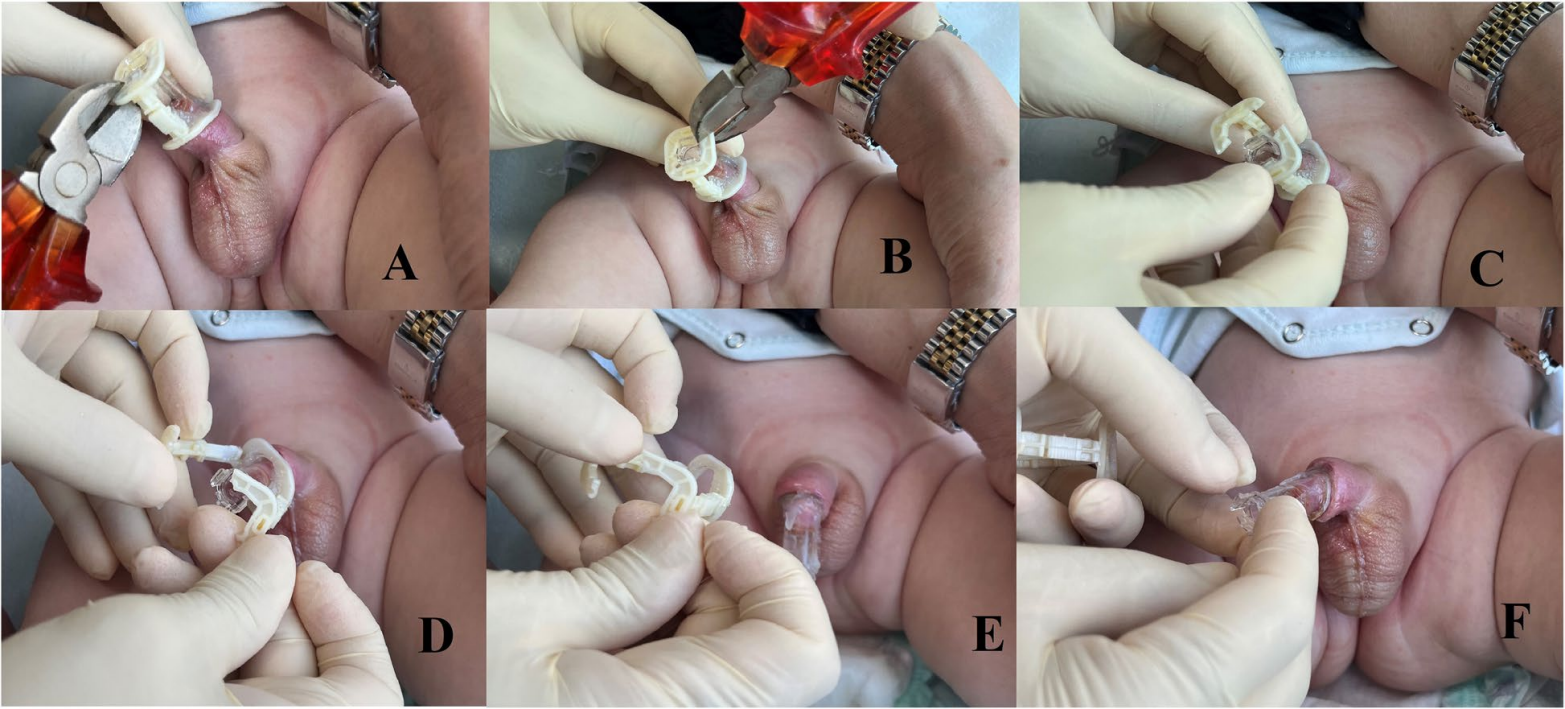
Steps of Sleeve circumcision. **A** Marking of the prepuce. **B** Marking of the inner mucosa. **C** Circumferential incision of the skin in a ring shape. **D** Circumferential incision of the inner mucosa. **E** Excision of the prepuce and inner mucosa together. **F** Hemostasis with bipolar cautery and closure of the skin with individual sutures for the final appearance after closure

Local wound care involved applying Combined Bacitracin and Neomycin Sulfate pomade (Thiocilline, Abdi İbrahim, İstanbul, Turkey) until clamp removal, typically on the fifth day postoperative. Removal of the clamp taked place 5–7 days after circumcision. The clamp was removed in the outpatient clinic without any anesthesia. After the plastic upper round part was cut with scissors, first the ring and then the glans protective part were removed. After clamp was removed, the use of local antibiotic cream was continued for another week (Fig. 2).

**Fig. 3:**
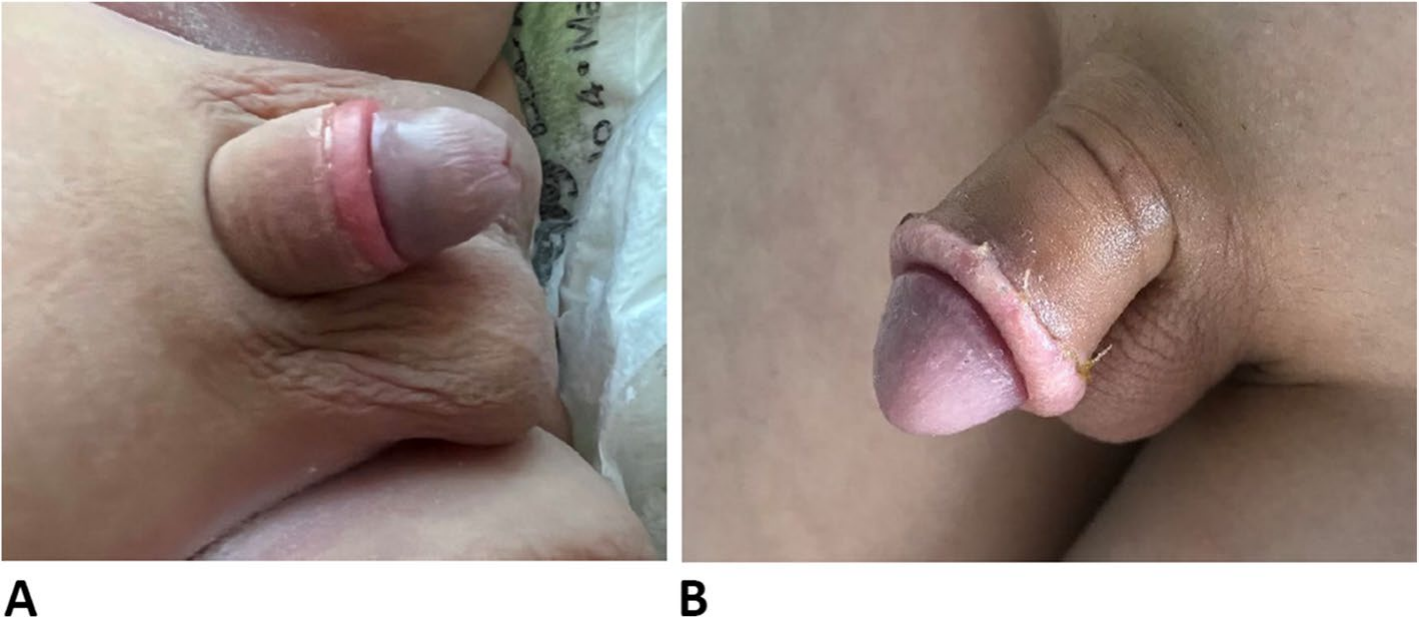
Appearance of circumcision performed with NeoAlis clamp and sleeve method after one month

One month after circumcision, patients underwent a follow-up examination to detect possible scarring and long-term complications. Figure 3 is one month after circumcision using the NeoAlis clamp.

#### Sleeve technique

**Fig. 4:**
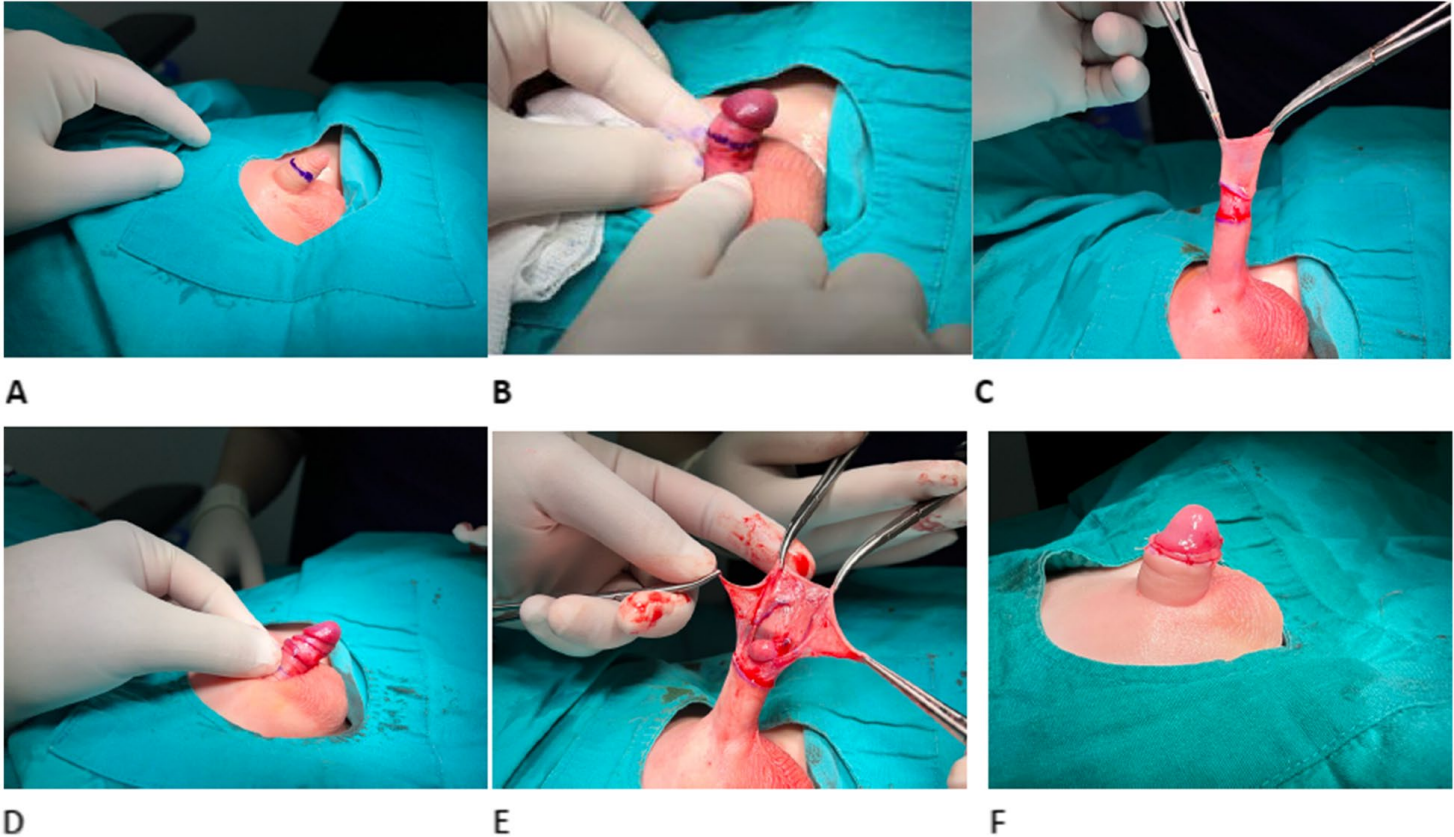
Steps of removing Neo Alis Clamp. **A** One edge of the outer ring is cut with the help of a side cutter. **B** The other edge of the opposite ring is cut. **C** The outer ring is removed from the inner protective cap. **D** The outer ring and the inner ring are completely separated from each other. **E** View of the inner ring after removing the outer ring. **F** The inner ring is removed

Procedures for local anesthesia were similar to those following general anesthesia, as with the NeoAlis clamp. External and internal preputial incisions were performed, and subcutaneous attachments surrounded by Buck’s fascia were separated. Excision of the prepuce and sleeve was done using scissors, while bipolar electro-cauterization was utilized to control bleeding. Closure was achieved using a 5 − 0 simple absorbable suture for mucosa and skin, as demonstrated in Fig. 4.

### Statistical analyses

The statistical analysis was performed utilizing the IBM SPSS 22.0 statistical software package (IBM Corporation, Chicago, IL, USA). Continuous variables were expressed as mean ± standard deviation and compared using the independent t-test. The Kolmogorov-Smirnov test was utilized to assess the normality of distribution. Student’s t-test was applied to compare normally distributed independent variables between the two groups. Statistical significance was defined as a *p*-value below 0.05, and *p*-values are reported to two significant digits, with any value less than 0.01 reported as “<0.01”.

## Results

**Fig. 5:**
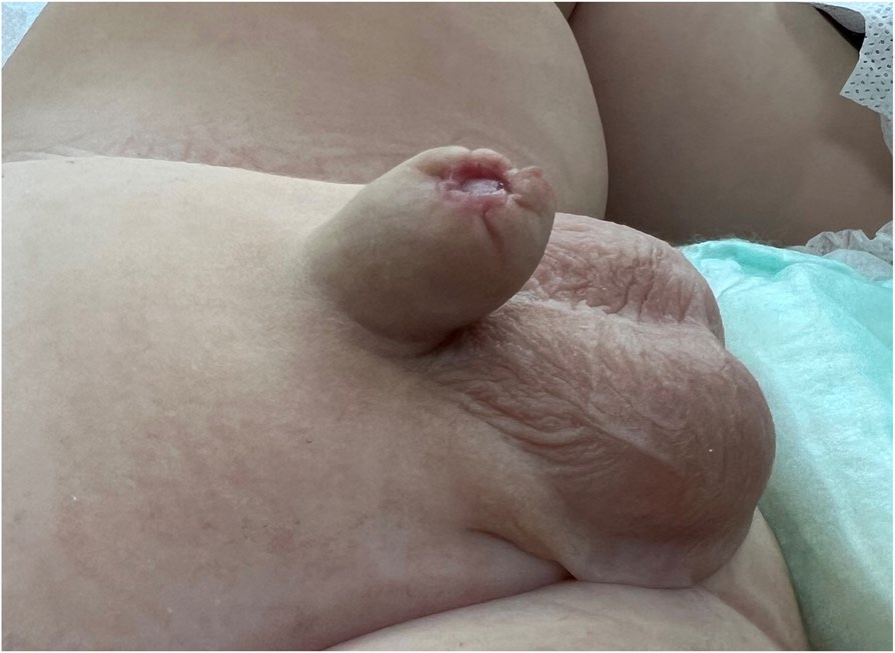
Secondary phimosis occurring after circumcision performed with the sleeve method

The research study included a cohort of 2626 patients, divided into two groups: Group 1 comprised 223 patients, and Group 2 comprised 2403 patients. The age range of the children involved spanned from 40 days to 16 years. Notable postoperative complaints included early-term pain and prolonged mucosal edema. Complications, excluding spontaneously ceasing oozing bleeding, occurred in 1.7% of cases (*n* = 47). Group 1 exhibited significantly shorter operation times. Table 1 summarizes the demographic and preoperative data of the entire cohort. On the other hand, Group 2, the rates of total bleeding, early adhesion, meatal stenosis, overall cost and surgical revision were notably elevated, with statistical significance (*p* < 0.005). A total of 46 children (2.2%) underwent revision surgery. Among them, 42 required surgical intervention due to bleeding. Four of them (two in group 1 and two in group 2) necessitated revision surgery due to secondary phimosis unresponsive to medical treatment (Fig. 5). Preputial revision was performed in these cases. Among the total, four patients developed wound site infections, three in the second group, and one in the first group. Meatal stenosis occurred in a patient who underwent traditional circumcision. This patient underwent urethral dilation twice and subsequently exhibited normal caliber urine flow. No cases of meatal stenosis were observed in circumcisions performed with the Neo Alis clamp. A wound dehiscence occurred in a patient undergoing traditional circumcision, managed conservatively with secondary healing, resulting in recovery without the need for further surgical intervention (Table 2). Post-circumcision complications are detailed in Table 2.

**Table 1:** Demographic and preoperative data

	Clamp (Group 1)	Classic (Group 2)	*P* value
Number	223	2403	
Age	4.2 ± 1.8 (month)	5.5 ± 3.1 (year)	0.001^*^
Obstructive phimosis	16	7	0.001^**^
Operation time (minutes)	7.3 ± 2.9	15.8 ± 6.9	0.001^*^
Prenatal hydronephrosis	21 (9.4%)	0	0.001^*^
Urinary tract infection	9	2	0.001^**^

*P* value is significant < 0.05

*: Student t test

**: Chie Square test

**Table 2:** Operative and postoperative data

	Clamp (Group 1)	Classic (Group 2)	*P* value
Number	223	2403	
Bleeding (Surgical intervention)	3 (1.3%)	34 (1.4%)	0.93^**^
Secondary phimosis (preputial residue)	2	2	0.003^***^
Wound separation	0	1	
Wound infection	1	3	0.23^**^
Urethral fistula	0	0	
Meatal stenosis	0	1	0.09^**^
Glandular injury	0	0	
Cost (Turkish liras)	296 ± 24	308 ± 15	0.001^*^

*P* value is significant < 0.05

*: Student t test

**: Chie Square test

^***^: Fisher’s exact test

The summary of the cost section is as follows: In terms of consumables, the cost of NeoAlis clamp and scalpel is 250 + 15 = 265 Turkish liras (TL). Together with local anesthesia and medical drugs used, this cost is estimated to be around 300 TL (approximately 10 US dollars as of 2024 exchange rates). The consumable cost in traditional surgical circumcision is seen as 5 − 0 polyglactin suture (Vicryl Rapid) and scalpel (70 + 15 = 85 TL). The use of anesthetic agents in traditional surgical circumcision averages 150 TL. There is no significant difference in total costs between the two groups (Table 2).

## Discussion

Despite its widespread practice, routine circumcision continues to be debated. On the other hand, many studies have attempted to determine the safest and least invasive surgical techniques for circumcision, a procedure motivated by both religious and medical concerns [17].

When deciding to perform circumcision, the three most important questions that arise are: What age should circumcision be performed, what should be the choice of anesthesia, and which surgical method should be preferred? As highlighted in this article, there are a large number of technical methods described, as well as a rapidly increasing number of auxiliary devices and disposable plastic rings [7]. A comparison was made between the most commonly preferred sleeve circumcision and the NeoAlis clamp, which is easy to learn and perform and is also rapid in terms of time [10, 11, 13, 14].

Although sleeve circumcision offers safety, excellent surgical outcomes, precise surgical control, and enhanced flexibility for surgeons, its adoption requires extensive attention and surgical training. However, due to factors such as the high volume of circumcision procedures, diverse environmental conditions, limited resources in densely populated areas, and the technical challenges involved, particularly in neonatal cases, the sleeve technique may not be a practical solution to meet the demand for circumcision in regions where it is routinely practiced for religious, traditional, or public health reasons [18, 19].

To meet the demand for a circumcision method that is both safe and effective, several devices and techniques have been developed. Among these, plastic clamp techniques, initially introduced with the PlastiBell, have gained popularity due to their simplicity, ability to secure the glans penis, and consistent cosmetic results [20]. Neo Alis clamp, an advancement of the smart clamp, is designed with a specific angle to focus on the ventral collar, while this clamp aims to preserve the frenulum while still serving as a reliable circumcision device, providing a stable platform for surgical procedures. Its two-piece design ensures complete protection of the glans penis and prevents any migration of the locking chamber. Following circumcision, the device can either be removed after a designated period or allowed to detach on its own. Lu et al. measured the frenulum lengths before and after surgery in 58 adult men and reported that the frenulum was preserved 100% in these patients using disposable devices [13]. When evaluating the short-term outcomes of circumcision techniques, previous studies have primarily concentrated on complications such as bleeding, injury to the glans penis or urethra, wound opening, wound infection, entrapment of the glans penis in the early postoperative period, necrosis of the glans penis, meatitis and/or meatal stenosis, early adhesions of the prepuce, unintentional injuries to the scrotal skin, and urinary retention. The rates of complications, techniques utilized, and the proficiency of practitioners vary considerably across the literature [21, 22].

The most feared complication of circumcision is damage to the glans [23], as it is difficult to reverse [24]. Our study findings reveal a notable absence of glans necrosis, glans penis injury, and scrotal injury in both experimental groups. It is noteworthy to highlight that the NeoAlis clamp, through its innovative two-piece design incorporating an inner tube, effectively mitigates the risk of glandular injury by isolating the glans penis from the incision plane. It can be said that there is no glans injury in circumcisions performed with NeoAlis clamp and other glans protective rings. The biggest advantage of these rings is that they protect the glans during circumcision [10–14, 25].

Bleeding after circumcision is the most common complication. Mano et al. reported the incidence of bleeding after circumcision as 0.32% [26]. There are also studies showing that routine bandaging after circumcision significantly reduces leakage bleeding [27]. Our investigation revealed bleeding rates necessitating surgical intervention consistent with literature norms (1.3% in group 1 and 1.4% in group 2), with no significant disparity between the groups. These rates align with acceptable standards for a high-volume community setting like ours. Bleedings that resolved spontaneously without surgical intervention were significantly higher in group 2; These were treated with simple surgical techniques (such as bandages). In circumcisions performed with NeoAlis, there is almost no annoying bleeding in the form of leakage. This can be considered an indicator of hemostatic effectiveness. One of the complications observed after circumcision is the formation of urethral fistula [28, 29]. In our study, we did not observe urethral fistula formation in any of the patients. Although varying rates of fistula formation have been reported in some studies, we believe that in recent years, due to better surgical and sterile conditions, this rate has decreased or disappeared. One of the potentially bothersome complications of circumcision is meatal stenosis, which typically occurs after newborn circumcision [30]. However, our only patient with this complication was 2 years old. Meatal stenosis was observed in one of the circumcisions performed using the sleeve method. This patient underwent urethral dilatation twice, after which normal voiding was observed.

One of the complications seen less frequently compared to previous years is wound site infection and the associated wound dehiscence [31]. According to our study results, wound infection was observed in one patient in group 1 and in three patients in group 2. Wound dehiscence occurred in one of the three patients in group two. Publications exist indicating that these rates are very low following circumcision performed under sterile conditions. Our study findings are consistent with the literature.

In this study, the total complication rate was lower compared to many studies in the literature [21]. This can be attributed to the patients being treated by the same senior surgeon at a single center, which wasn’t a training clinic. Additionally, since 2005, the Ministry of Health’s ban on home circumcision and on circumcision performed by medical personnel has led to a decrease in complications observed in Turkey [32].

One of the most frequently debated topics during circumcision is the speed at which the procedure can be performed. In countries like Turkey where routine traditional circumcision is performed in large numbers, some clinics may face pressure to accommodate circumcision demand within their working hours [12, 25]. As in our study, circumcision using the NeoAlis clamp is significantly faster than traditional circumcision because there is no need for bleeding control or suturing. This increased speed allows for the demand to be met more rapidly.

Since the patient is under anesthesia, the classical technique involves additional costs for both anesthetic drugs and personnel. In contrast, the technique performed with the NeoAlis Clamp is local, eliminating anesthesia-related expenses. Therefore, the cost is lower in Clamp-assisted circumcision.

There are some limitations related to this study. The number of patients included in the study is quite sufficient for evaluation, but patient selection is heavily dependent on age. Circumcisions performed with the NeoAlis clamp encompass children under one year of age. Although it is technically feasible to perform the procedure on patients over one-year-old, older children often experience discomfort from wearing a plastic ring around the penile glans for five days. In Turkey and similar countries, routine circumcision for religious and cultural purposes is commonly performed. This circumcision procedure is often carried out as part of a wedding ceremony, and consequently, some families prefer to have the circumcision done shortly before the child starts school, during the period just before school enrollment. Therefore, in order to avoid the discomfort caused by the plastic ring in older children’s circumcisions, conventional circumcision has been preferred.

## Conclusion

In this study, we aimed to compare the outcomes of two commonly used techniques. While the sleeve method is widely practiced, disposable circumcision assisting devices like the NeoAlis clamp have gained popularity in recent years. Learning and using the NeoAlis clamp for circumcision is straightforward. It is quick and safe since steps involving bleeding control and suturing are skipped. The main point we want to emphasize in this study is that for infants under one year old needing circumcision and where avoiding general anesthesia is preferred, the NeoAlis clamp is a safe and rapid option.

### Supplementary Information

Additional file 1: Video 1: Circumcision with Neo Alis Clamp. In this video, all stages of circumcision performed with the help of Neo Alis can be watched. After local anesthesia, sterile dye was applied to the 2-month-old male baby and a sterile drape was applied. Then circumcision was performed.

## Data Availability

The datasets generated and analysed during the current study are not publicly available due to patients’ privacy but are available from the corresponding author on reasonable request.
