# Correction: Comparative analysis of two methods in circumcision: a new disposable device versus classic sleeve technique

**DOI:** 10.1186/s12894-024-01554-0

**Published:** 2024-07-31

**Authors:** Sinan Kılıç

**Affiliations:** Department of Pediatric Surgery, Private Gebze Yuzyil Hospital, Clinic of Pediatric Surgery, Gebze, Kocaeli Turkey

**Correction to: BMC Urology (2024) 24:126**

10.1186/s12894-024-01513-9

Following publication of the original article [1], Figs. 4 and 5 captions had been interchanged. Caption of Fig. 5 inadvertently give in as caption for Fig. 4 and vice versa. The figure(s) should have appeared as shown below.

**Fig. 4:**
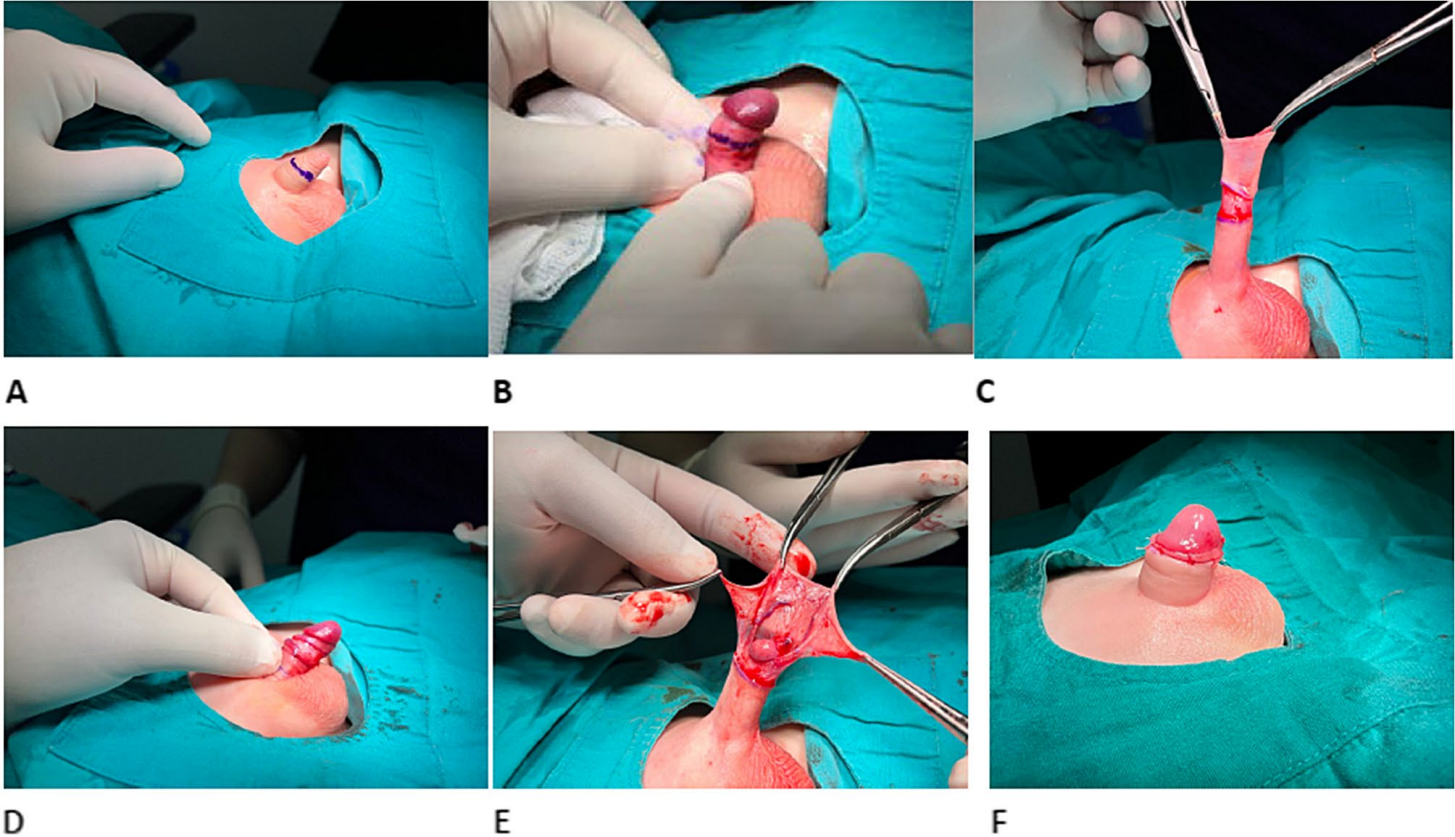
Steps of removing Neo Alis Clamp. **A** One edge of the outer ring is cut with the help of a side cutter. **B** The other edge of the opposite ring is cut. **C** The outer ring is removed from the inner protective cap. **D** The outer ring and the inner ring are completely separated from each other. **E** View of the inner ring after removing the outer ring. **F** The inner ring is removed

**Fig. 5:**
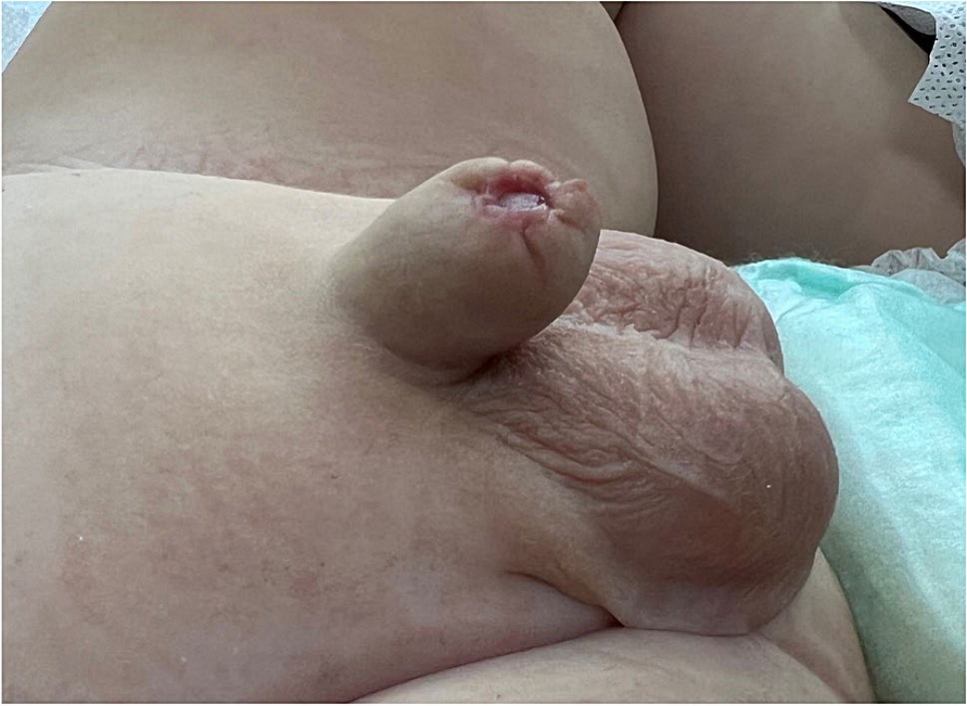
Secondary phimosis occurring after circumcision performed with the sleeve method

The original article has been corrected.
